# Evaluation of effect and safety of arthroscopic surgery with three different operative approaches in patients with terrible triad of the elbow: A comparative study

**DOI:** 10.1097/MD.0000000000010398

**Published:** 2018-04-13

**Authors:** Tao Li, Xing-Long Li, Shi-Xiang Hu, Wei Sun, Jing Wu

**Affiliations:** a Department of Emergency and Trauma Surgery, Jining No.1 People's Hospital, Jining; b Department of Orthopedics, Yankuang Group Genaral Hospital, Zoucheng; c Department of Surgery, Xizou Health Center of Qufu, Qufu; d Department of Surgery, Taiping Town Health Center of Zoucheng, Zoucheng, P.R. China.

**Keywords:** arthroscopic surgery, effect, operative approaches, postoperative complications, terrible triad of the elbow

## Abstract

**Background:**

The terrible triad of the elbow (TTE) is a difficult injury, and the usual TTE consists of posterior dislocation of the elbow, radial head fracture, and coronoid fracture. The target of this retrospective study is to explore the effect, postoperative complications, and prognostic factors in patients with TTE undergoing arthroscopic surgery with three different operative approaches.

**Methods:**

Patients with TTE underwent arthroscopic surgery were treated using lateral, anterior medial, or combined lateral and anterior medial approaches, respectively. In order to analyze the postoperative complications and the effect of arthroscopic surgery for patients with TTE, the function of elbow joint before and after the surgery was evaluated and the flexion-extension of elbow joint and rotation of elbow joint and forearm were measured.

**Results:**

The evaluation results obtained from patients underwent 3 different operative approaches revealed that after surgery, patients receiving arthroscopic surgery using combined lateral and anterior medial approach had superior flexion-extension of elbow joint, rotation of elbow joint and forearm, higher Mayo Elbow Performance Score (MEPS) and more cases of Broberg–Morrey grade 0, and lower complication rate, compared with those using lateral or anterior medial approach. A key finding in the study demonstrated that fracture type, operative approach, and postoperative complications were factors related with the effect of arthroscopic surgery for patients with TTE.

**Conclusion:**

Collectively, the key findings obtained from the present study supported the notion that the effect of arthroscopic surgery in combined lateral and anterior medial approach is much better than in lateral approach or anterior medial approach, and is related with fracture type, operative approach, and postoperative complications.

## 1 Introduction

Terrible triad of the elbow (TTE) is universally known for the dislocation of the elbow, fracture of radial head, and ulnar coronoid fracture.^[1]^ The injury often results in instability of elbow and is recognized as a most challenging injury in the musculoskeletal system.^[2]^ Young male patients might suffer from TTE in general, which is associated with high-energy trauma.^[3]^ Supination, valgus stress, falling onto the hand, and elbow under hyperextension make up the common mechanism for TTE.^[4]^ They are responsible for 4% adult fractures of radial head and 31% elbow dislocations.^[5]^ Conservative or nonoperative management usually demands close clinical and radiographic follow-up, in order to keep monitoring any possible delay of fracture displacement or elbow subluxation.^[6]^ Traditional nonoperative method has been proven poor in the treatment of TTE due to repetitive instability and stiffness induced by long-term fixation, and operative treatment for TTE is advocated at present.^[7,8]^ Heterotopic ossification is a common complication follows elbow fracture surgery, which can significantly impair the range and function of motion.^[9]^ Hence, it is of great value to have a research on the efficacy of operative approaches in TTE.

Elbow lateral approach facilitates the repairing of radial head fracture and lateral collateral ligaments, while anterior medial approach acts to repair anterior joint capsular, coronal head and injuries of the internal collateral ligaments, and lateral and posterior approaches are widely adopted for TTE nowadays.^[10]^ Anterior medial approach is used to stabilize coronoid fractures by using buttress plating together with anterior to posterior screw fixation, which permitted stable internal fixation and anatomic reduction during follow-up in the near future.^[11]^ The most common complication following medial collateral ligament surgery is ulnar neuropathy.^[12]^ Rodriguez-Martin et al^[4]^ demonstrated that posterolateral and anterior medial approaches were effective in treating TTE. According to another study, patients with TTE undergoing combined lateral and anterior medial approach have more effective results than posterior approaches.^[13]^ Combined lateral and medial approach is fit for complex fracture patterns, but it is reported to have a high complication rate.^[14]^ Different operative approaches have merits and demerits, and therefore a right way of treatment in TTE remains to be decided. The study was to evaluate the efficacy, postoperative complications, and prognostic factors of lateral, anterior medial, and combined lateral and medial approaches in TTE treatment.

## 2 Materials and methods

### 2.1 Ethics statement

This study was approved by the ethics committee of Jining No.1 People's Hospital. Written informed consents were obtained from all study subjects.

### 2.2 Study subjects

Between May 2008 and May 2013, a total of 261 patients with closed TTE underwent arthroscopic surgery in Jining No.1 People's Hospital were selected into the study. There were 170 males and 91 females with an average age of 39.72 ± 3.96 years (range from 28 to 51 years). On average 4 days after injury (range from 2 to 8 days), they received arthroscopic surgery. Among the 261 included subjects, 88 cases underwent surgery by lateral approach, 92 by anterior medial approach, and 81 received combined lateral and anterior medial approach. All participants met diagnostic criteria of TTE.^[7]^ All the patients were diagnosed with unilateral fracture, 126 with TTE of left, 135 with TTE of right. The causes of injury included 66 from falls, 40 from traffic accidents, and 155 from intense collisions. According to the Mason typing of radial head classification^[15]^: 62 patients (23.75%) were in type I (marginal fracture without bone block displacement, which was reconstructable); 128 patients (49.04%) in type II (fracture with bone block displacement, which was reconstructable); and 71 patients (27.20%) in type III (comminuted fracture, which was unreconstructable). On the basis of the Regan–Morrey typing of ulna coronoid process of fracture classification,^[16]^ they were classified as following: type I, 75 patients (28.74%) with avulsion fracture of the coronoid process; type II, 116 patients (44.44%) with a fracture no more than 50% of the entire coracoid process; and type III, 70 patients (26.82%) with a fracture more than 50% of the entire coracoid process.

### 2.3 Surgical treatment

After the patients were admitted to hospital, the frontal and lateral X-ray, computed tomography (CT) scan, and 3-dimensional reconstruction were used to determine the type of injury. Together with physical examination and electrocardiogram, the patients were then asked if they had other diseases or not and their tolerance was evaluated. During medical emergencies, manual reduction and plaster fixation were used for dislocation of elbow. When giving operative approaches, lateral approach was used for radial head replacement, and combined lateral and anterior medial approach for Regan–Morrey type III fracture, and anterior medial approach for the rest of the patients.

During operation, after brachial plexus block anesthesia, the patients were placed in supine position with the limb on the side table. For lateral approach: an incision was made between triceps and brachioradialis muscle along the lateral epicondyle of the humerus, with an extension between the anconeus and extensor carpi ulnaris to expose the lateral collateral ligament, the joint capsule, and the radial head. For anterior medial approach: an incision was made between flexor carpi radialis and palmaris longus by musculus flexor digitorum superficialis. For the combined approach: an incision was processed between triceps and brachioradialis muscle group along the lateral epicondyle of the humerus, with an extension between the anconeus and extensor carpi ulnaris for the sake of exposing the joint capsule and the lateral collateral ligament. And an incision was made between flexor carpi radialis and palmaris longus by flexor digitorum superficialis as well. Regan–Morrey type I fracture underwent internal fixation with tension band, type II to III fracture was fixed by screw fixation. Internal fixation with Kirschner wire, 3.5 mm hollow screw, and T-shaped plate was used for patients with type I to II fracture of radial head, and radial head replacement was applied for patients with type III fracture. After surgery, the patients lay in bed with the elbow bent at 90° and the forearm in neutral position. The elbow was hung in chest position by arm sling. The patients was taken good care of after brachial plexus block anesthesia and were given continuous oxygen inhalation and monitoring of vital signs. Medicines to promote blood circulation and reduce swelling as well as of orthopedics-traumatology coaptation were applied for fracture healing. After surgery, movement and physical condition of patients were observed, and the stitches were then taken out 4 weeks later.

### 2.4 Follow-up and complication records

A follow-up was carried out in all the patients, for 24 months on average (range from 12 to 36 months). The patients were rechecked monthly after surgery until complete recovery. The recheck mainly involved frontal and lateral X-ray, and rotation of forearm to evaluate the stability of elbow, and joint fusion, heterotopic ossification, traumatic arthritis, or other complications were recorded.

### 2.5 Efficacy evaluation

The elbow joint function including elbow stability, activity, and pain was evaluated on the basis of Mayo Elbow Performance Score (MEPS).^[17]^ The full score was 100 points. A score over 90 points was recognized excellent, 75 to 89 points as good, 60 to 74 points as acceptable, and lower than 60 points as poor. The flexion-extension of elbow joints and rotation of elbow joints and forearm were measured. X-ray images were analyzed and loosening of internal fixation, fracture reduction, heterotopic ossification, and complications were recorded, and complication rate was calculated. The Broberg–Morrey classification was used to evaluate the degree of degenerative changes of the elbow. Grade 0 was normal joint; grade 1 was slight joint space narrowing accompanied by a small amount of osteophyma; grade 2 was moderate degree of joint space narrowing accompanied by some osteophyma; and grade 3 was serious degenerative change accompanied by joint destruction.

### 2.6 Statistical analysis

All the data were analyzed by SPSS 19.0. Measurement data were presented as mean ± standard deviation. Comparison between 2 groups was analyzed by *t* test and among multiple groups by variance analysis. Enumeration data are showed as percentage or rate and were compared by chi-square test. *P* value <.05 was considered statistically significant. Logistic regression analysis was established to conduct multi factor analysis.

## 3 Results

### 3.1 Comparison of baseline characteristics of patients undergoing 3 different operative approaches indicates no statistical difference

Initially, the basic data of patients and 3 different operative approaches are shown in Table 1, and the comparison showed there were no significant differences in age, gender, fracture causes, surgical sites, or fracture type among patients undergoing lateral, anterior medial, and combined lateral and anterior medial approaches (all *P* > .05).

**Table 1:**
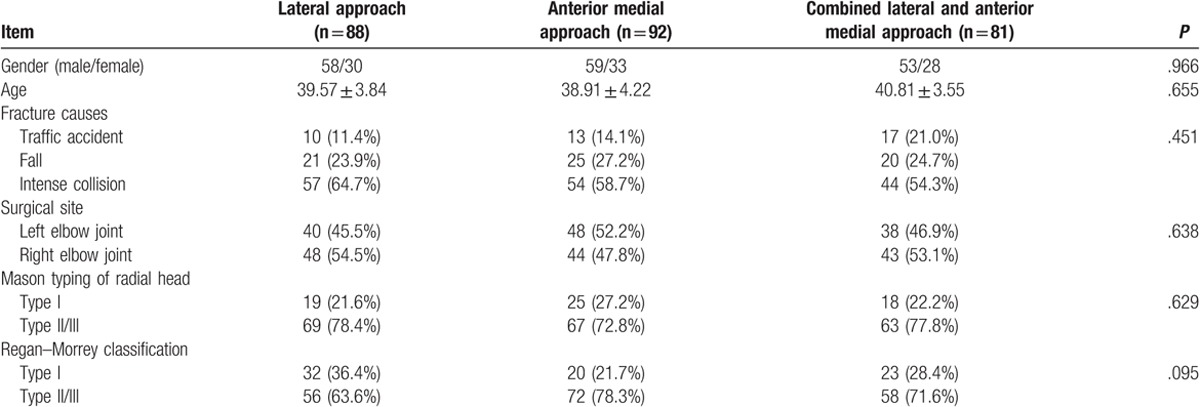
Baseline characteristics of patients undergoing 3 different operative approaches.

### 3.2 Patients with TTE achieve good fracture healing after undergoing 3 different operative approaches

**Figure 1:**
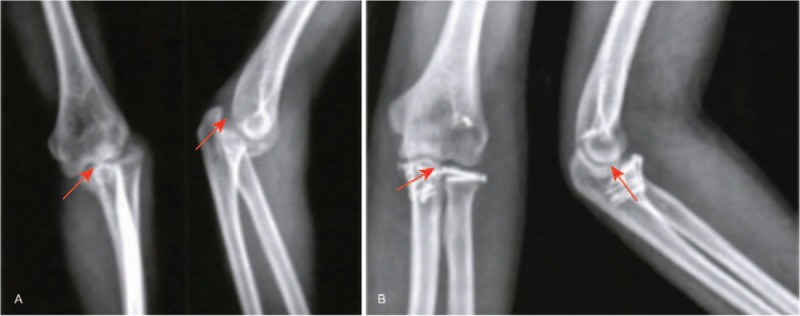
Frontal and lateral X-ray of the elbow joint in a terrible triad of the elbow (TTE) patient caused by a traffic accident before and after surgery, male patient, 42 years old, terrible triad of the right side of the elbow was caused by a traffic accident; the combined lateral and anterior medial approach was used. (A) Frontal and lateral X-ray images before surgery; (B) frontal and lateral X-ray images 6 months after surgery. The red arrow in the figure indicated the injury part.

**Figure 2:**
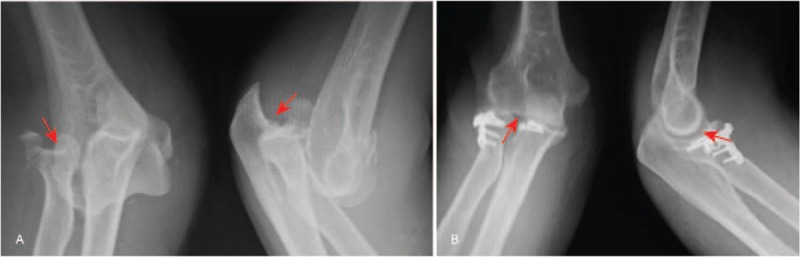
Frontal and lateral X-ray of the elbow joint in a terrible triad of the elbow (TTE) patient caused by fall before and after surgery, female patient, 35 years old, terrible triad of the right side of the elbow was caused by fall; the lateral approach was used. (A) Frontal and lateral X-ray images before surgery; (B) frontal and lateral X-ray images after surgery. The red arrow in the figure indicated the injury part.

**Figure 3:**
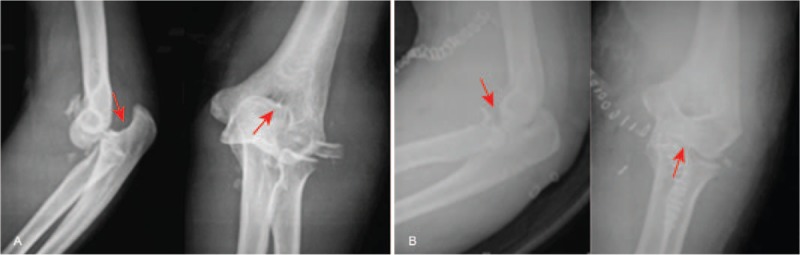
Frontal and lateral X-ray of the elbow joint in a terrible triad of the elbow (TTE) patient caused by intense collision before and after surgery, female patient, 51 years old, terrible triad of the right side of the elbow was caused by intense collision; the anterior medial approach was used. (A) Frontal and lateral X-ray images before surgery; (B) frontal and lateral X-ray images 3 months after surgery. The red arrow in the figure indicated the injury part.

In order to observe the effect of 3 operative approaches, X-ray images were obtained. X-ray for representative patients undergoing 3 different operative approaches in Figs. 1–3 revealed that compared with before surgery, in patients after surgery, fracture improved a lot, elbow joint was in stable, fracture site healed gradually, and fracture line also disappeared gradually. Therefore, the conclusion was reached that after undergoing 3 different operative approaches, all patients achieved good fracture healing.

### 3.3 Patients underwent the combined lateral and anterior medial approach display the best efficacy and recovery

In order to compare the efficacy and recovery of patients undergoing 3 different operative approaches, the data of flexion-extension of elbow joint, rotation of elbow joint, and forearm were compared. Results showed that in patients using lateral, anterior medial, and combined lateral and anterior medial approaches, the flexion-extension of elbow joint, rotation of elbow joint, and forearm all improved. Importantly, the recovery of the patients receiving arthroscopic surgery using combined lateral and anterior medial approach was much better than those with lateral and anterior medial approaches (both *P* *<* .05) (Table 2).

**Table 2:**
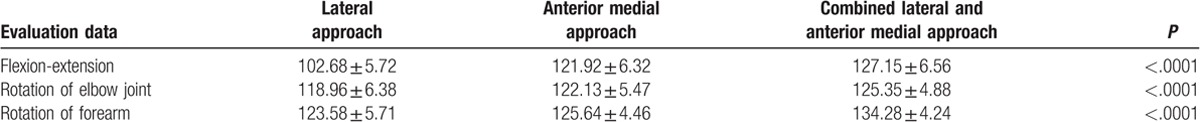
Comparisons of efficacy and recovery of patients undergoing 3 different operative approaches.

### 3.4 Patients underwent the combined lateral and anterior medial approach have the highest MEPS

MEPS was applied to compared the effect of 3 different operative approaches. As shown in Table 3, the results demonstrated that in patients undergoing combined lateral and anterior medial approach, MEPS was excellent after surgery in patients undergoing 3 different operative approaches, and the difference was statistically significant compared with before surgery (all *P* < .05). Besides, the scores of anterior medical and lateral approaches were not statistically significant (*P* *>* .05). Lastly, an important finding revealed that the MEPS for patients with combined lateral and anterior medial approach was significantly higher than those with lateral and anterior medical approaches after surgery (both *P* < .05).

**Table 3:**
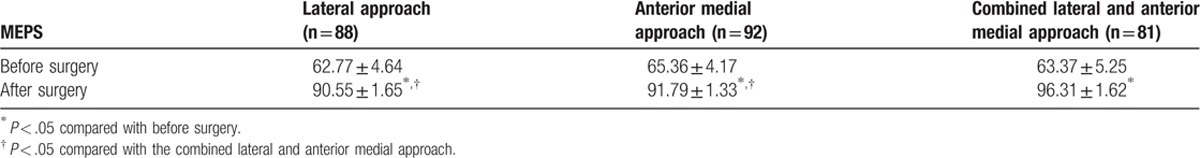
Comparisons of Mayo Elbow Performance Score (MEPS) before and after surgery of patients undergoing 3 different operative approaches.

### 3.5 The combined lateral and anterior medial approach has the best efficacy to recover the function of elbow joint

Broberg–Morrey classification was applied to evaluate the case of patients who have recovered the function of elbow joint. The results demonstrated that the number of patients undergoing combined lateral and anterior medial approach in Broberg–Morrey grade 0 (normal joint) was obviously higher than that of patients undergoing lateral and anterior medial approaches (both *P* < .05) (Table 4). Therefore, the combined lateral and anterior medial approach had the most cases that recovered the function of elbow joint.

**Table 4:**
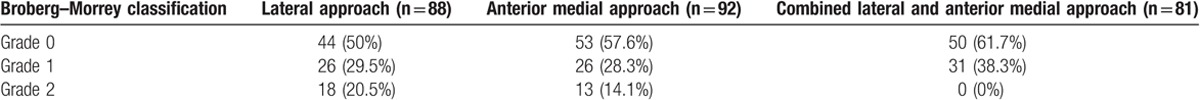
Broberg–Morrey classification of degenerative changes in patients undergoing 3 different operative approaches.

### 3.6 Patients undergoing the combined lateral and anterior medial approach have less postoperative complications

Postoperative complications of patients underwent 3 different operative approaches were compared and the complications involved heterotopic ossification, wound infection, ulnar nerve palsy, dislocation of elbow, and joint stiffness (Table 5). Then the comparison results indicated that patients undergoing combined lateral and anterior medial approach (6.17%) had a lower complication rate than those undergoing lateral (13.64%) and anterior medical approaches (13.04%) (both *P* < .05). Therefore, patients who underwent the combined lateral and anterior medial approach had less postoperative complications.

**Table 5:**
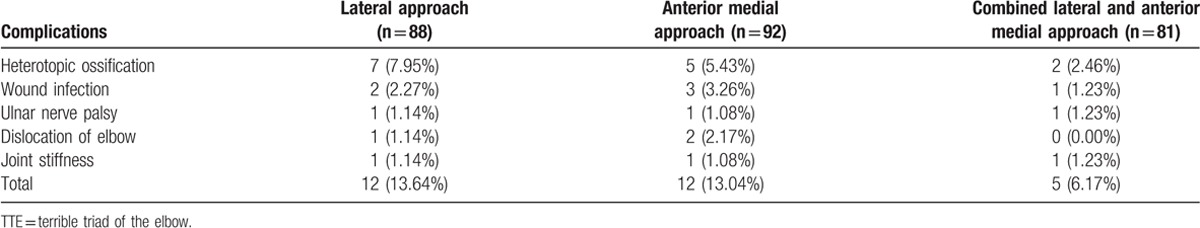
Complication rate of TTE patients undergoing 3 different operative approaches.

### 3.7 Arthroscopic surgery efficacy is related with Regan–Morrey classification, operative approach, and postoperative complication

Lastly, in order to evaluate the arthroscopic surgery efficacy-related factors, the logistic regression analysis was established with MEPS (>90) as dependent variable, and gender, surgical site, fracture type, operative approach, and postoperative complication were put in the model for multifactor analysis. Then the results indicated that the effect of arthroscopic surgery was related with Regan–Morrey classification, operative approach, and postoperative complication (all *P* < .05) (Table 6).

**Table 6:**
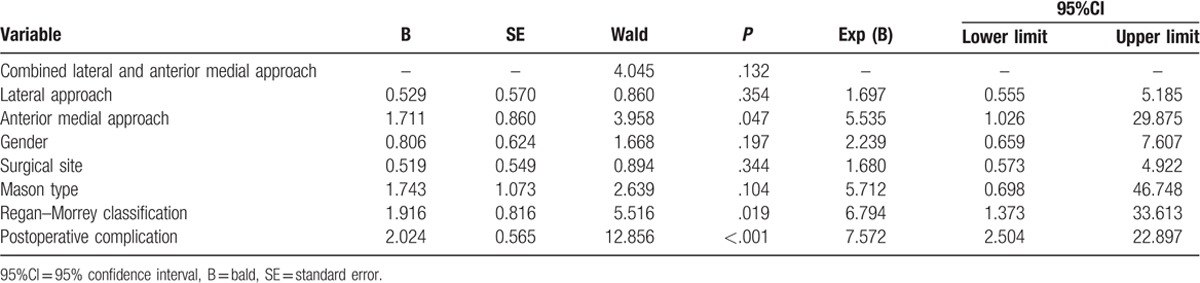
Logistic regression analysis of effect of arthroscopic surgery for patients undergoing 3 different operative approaches.

## 4 Discussion

Ulna coronoid process, radial head fractures, and elbow joint's posterior dislocation are the characteristics of TTE,^[10]^ which can bring about serious instability of elbow. For the sake of restoring the anatomic structures, repairing the articular capsule, and the collateral ligament, surgery should be used, in conjunction with the adjuvant hinged external fixation and some early exercise, to prevent immobilization and recover the function of elbow.^[18]^ Therefore, there is a pressing need to find a proper and efficient surgical approach for TTE. In this study, we concentrated on 3 different operative approaches, lateral, anterior medial, or combined lateral and anterior medial approaches, and we found that compared with TTE patients underwent lateral approach or anterior medial approach, patients using combined lateral and anterior medial approach exhibited a better effect of treatment.

After undergoing 3 different operative approaches, all patients were in good condition. Compared with before surgery, in all included patients after surgery, fracture improved a lot, elbow joint was in stable, fracture site healed gradually, and fracture line also disappeared gradually. Moreover, it can be seen that the function and range of motion showed some improvement. The flexion-extension and rotation of elbow joint, and rotation of forearm all improved. Our results indicated the great merit of operative approach for TTE patients. The traditional method for TTE treatment was found to contribute to a comparatively rising dislocation rate due to persistent instability.^[19]^ Although the surgical treatment allows early motion postoperatively, restores elbow stability sufficiently, promotes the functional outcome, and should therefore be recommended to restore the collateral ligament, to refrain from immobilization, to repair the anatomic structures, and to rehabilitate articular function.^[20]^ Besides, our results revealed that patients using combined lateral and anterior medial approach recovered better after surgery, in comparison to patients with the other 2 approaches. Anterior medial approach is favorable to clear exposure, small invasion, placement of internal fixation, and good clinical results; it also plays an active role in reducing and fixing coronoid process fractures.^[21]^ Lateral approach begins with the starting point on the lateral condyle of the humeral in order to protect or release the lateral collateral ligament, and then the elbow can be dislocated and part of the trochlear and the capitellum can be exposed.^[22]^ Combined lateral and anterior medial approach makes the full use of the strength of the 2. Chen and Bi^[1]^ demonstrated that combined lateral and anterior medial approach leads to extended surgical exposure, fracture stability, and reduced complication rate, therefore demonstrated better curative effects than the other 2 surgical approaches. Du and Zhu^[17]^ showed that through combined lateral and anterior medial approach, surgical treatment for TTE favors in allowing early exercise, providing stability in bone and soft-tissue, and at the same time, ameliorating functional recovery at early stages.^[17]^ As it should be, the postoperative outcome of the patients with arthroscopic surgery using combined lateral and anterior medial approach was superior to those with lateral and anterior medial approaches.

Markedly higher MEPS was shown in TTE patients receiving the combined lateral and anterior medial approach than those undergoing lateral or anterior medical approaches. MEPS is highly applied to evaluate disability of elbow injuries.^[23]^ A recent study showed that restoring the damaged structures, such as medical soft tissue structures, demonstrates excellent results on the basis of MEPS in TTE patients.^[24]^ By using a single anterior approach in a study with 79 patients undergoing surgical treatment of distal biceps tendon ruptures with anterior bone anchor, the MEPS was 95.2 points and nearly every patient had excellent and good results.^[25]^ As Kälicke et al^[26]^ found, regaining excellent function of stable joint rests with early exercise-stable immobilization, reestablishment of coronoid process, as well as mobilizing the joint functionally in the early stage.

In this study, our data of the Broberg–Morrey classification in TTE patients using one of the 3 approaches showed that in Broberg–Morrey grade 0 (normal joint), there were exceedingly more patients receiving the combined lateral and anterior medial approach than those undergoing the other 2 kinds. Broberg–Morrey classification was also used to evaluate preoperative and postoperative elbow functions in a study about osteochondral transplantation.^[27]^ Good or excellent results were achieved in Mason type II and type III fractures using the Broberg–Morrey score.^[28]^ Zhang et al^[29]^ showed that the efficacy of patients with capitellar fractures undergoing different operative approaches were evaluated with Broberg–Morrey score system, the average score was 92.5 points (range: 62–100 points), and the excellent and good rate was 91.8%.

In addition, patients undergoing combined lateral and anterior medial approach exhibited lower complication rate compared with those with the other 2 approaches. Complications after surgical treatments include synostosis, displacement, reducing secondary loss, and necrosis of radial head.^[30]^ In consistent with our study, a recent research also discovered that combined lateral and anterior medial approach might remain the best approach for TTE patients and in comparison with anterior medial approach, a remarkably lower complication rate was found in patients using combined lateral and anterior medial approach.^[1]^

In summary, our study found that patients using combined lateral and anterior medial approach presented a better curative effect for the treatment of TTE compared with patients receiving lateral or anterior medial approach. However, the reliability of our findings was limited due to limited sample size and insufficient ability to distinguish significant differences. Further exploration for specific mechanisms of the joint treatment is demanded in future studies with larger sample size.

## Acknowledgments

The authors thank the reviewers for critical comments.

## Author contributions

**Conceptualization:** T. Li, X-L. Li, S-X. Hu, J. Wu.

**Data curation:** T. Li, X-L. Li, S-X. Hu, W. Sun, J. Wu.

**Formal analysis:** T. Li, X-L. Li, S-X. Hu, W. Sun, J. Wu.

**Investigation:** T. Li, X-L. Li, S-X. Hu, W. Sun, J. Wu.

**Methodology:** T. Li, X-L. Li.

**Project administration:** T. Li, X-L. Li, S-X. Hu, W. Sun, J. Wu.

**Resources:** T. Li, X-L. Li, S-X. Hu, W. Sun.

**Software:** T. Li.

**Supervision:** T. Li, X-L. Li, S-X. Hu, W. Sun, J. Wu.

**Validation:** T. Li, X-L. Li, S-X. Hu, W. Sun, J. Wu.

**Visualization:** T. Li, X-L. Li, S-X. Hu, W. Sun.

**Writing – original draft:** T. Li, X-L. Li, S-X. Hu, W. Sun, J. Wu.

**Writing – review and editing:** T. Li, X-L. Li, S-X. Hu, W. Sun, J. Wu.
